# Change in circulating klotho in response to weight loss, with and without exercise, in adults with overweight or obesity

**DOI:** 10.3389/fragi.2023.1213228

**Published:** 2023-06-30

**Authors:** Katherine A. Collins, Fabrisia Ambrosio, Renee J. Rogers, Wei Lang, Eric B. Schelbert, Kelliann K. Davis, John M. Jakicic

**Affiliations:** ^1^ Duke Molecular Physiology Institute, Duke University School of Medicine, Durham, NC, United States; ^2^ Discovery Center for Musculoskeletal Recovery, Schoen Adams Research Institute at Spaulding, Boston, MA, United States; ^3^ Department of Physical Medicine and Rehabilitation, Harvard Medical School, Boston, MA, United States; ^4^ Department of Internal Medicine, University of Kansas Medical Center, Kansas City, KS, United States; ^5^ Department of Aging Medicine and Center on Aging and Mobility, University Hospital Zurich and University of Zurich, Zurich, Switzerland; ^6^ School of Medicine, University of Pittsburgh, Pittsburgh, PA, United States; ^7^ Minneapolis Heart Institute East, Saint Paul, MN, United States; ^8^ Department of Health and Human Development, University of Pittsburgh, Pittsburgh, PA, United States

**Keywords:** aging, klotho, obesity, physical activity, weight loss

## Abstract

**Introduction:** Klotho is a protein associated with protection from aging-related diseases and health conditions. Obesity is associated with lower Klotho concentrations. Thus, this secondary analysis of adults with obesity examined 1) the change in serum Klotho concentration in response to a behavioral weight loss intervention by the magnitude of weight loss achieved; and 2) the association among serum Klotho concentration and weight, body composition, and cardiorespiratory fitness.

**Methods:** Participants were randomized to either diet alone (DIET), diet plus 150 min of physical activity per week (DIET + PA150), or diet plus 250 min of physical activity per week (DIET + PA250). Participants [*n* = 152; age: 45.0 ± 7.9 years; body mass index (BMI): 32.4 ± 3.8 kg/m^2^] included in this secondary analysis provided blood samples at baseline, 6-, and 12 months, and were classified by weight loss response (Responder: achieved ≥10% weight loss at 6 or 12 months; Non-responder: achieved <5% weight loss at both 6 and 12 months). Serum Klotho was measured using a solid-phase sandwich enzyme-linked immunosorbent assay (ELISA). Analyses of covariance (ANCOVA’s) were used to examine changes in weight, body composition, cardiorespiratory fitness, and Klotho concentration by weight loss response across the 12-month weight loss intervention.

**Results:** Responders had a greater reduction in measures of weight and body composition, and a greater increase in cardiorespiratory fitness, compared to Non-Responders (*p* < 0.05). Change in Klotho concentration differed between Responders and Non-Responders (*p* < 0.05), with the increase in Klotho concentration from baseline to 6 months for Responders being statistically significant. The 6-month change in Klotho concentration was inversely associated with the 6-month change in weight (*r*_s_ = −0.195), BMI (*r*_s_ = −0.196), fat mass (*r*_s_ = −0.184), and waist circumference (*r*_s_ = −0.218) (*p*-values <0.05).

**Discussion:** Findings provide evidence within the context of a behavioral intervention, with and without exercise, that change in Klotho concentration is significantly different between adults with weight loss ≥10% compared to <5% across 12 months. These findings suggest that weight loss and reduction in fat mass may be favorably associated with the change in Klotho concentration. This may reduce the risk of negative health consequences associated with accelerated aging in middle-aged adults.

## Introduction

Overweight and obesity are associated with major health risks and increased risk for premature death (Van Gaal et al., 2006; Hales et al., 2017). The current prevalence of obesity in the United States is approximately 41.9% (Stierman et al., 2021). Due to obesity’s association with many chronic diseases such as cardiovascular disease, diabetes, and certain cancers, among others, the increased prevalence of obesity is of significant public health concern (Must et al., 1999; Vgontzas et al., 2000; Field et al., 2001; Mokdad et al., 2003; Wolk et al., 2003; Poirier et al., 2006). These health-related concerns of obesity may be a result of excess body weight affecting aging mechanisms through the maturation of adipose tissue, influencing inflammation and glucose homeostasis, oxidative stress, DNA damage, telomere dysfunction, and increased vasomotor tone and sympathetic drive (Seidell et al., 1992; Steinberg et al., 1996; Weinsier et al., 1998; He et al., 2001; Woo et al., 2004; Poirier et al., 2006; Ahima, 2009; Consitt et al., 2009; Galgani and Ravussin, 2009; Barton, 2010; Flegal et al., 2012; Hales et al., 2017). The accelerated aging processes that result from overweight and obesity may serve as a mechanistic pathway leading to the development of chronic diseases.

One biomarker, among others, that may provide insight into the accelerated aging process and accompany obesity is Klotho (Orces, 2022a; Orces, 2022b). Klotho is a protein shown to promote longevity and provide cardiovascular and neuroprotective effects (Arking et al., 2003; Wang and Sun, 2009; Semba et al., 2011; Razzaque, 2012; Semba et al., 2012; Drüeke and Massy, 2013; Avin et al., 2014; Di Bona et al., 2014; Semba et al., 2015; Xu and Sun, 2015; Hui et al., 2017). Circulating Klotho results from either direct secretion by the cell or from cleavage of the intracellular domain of the full-length protein by secretases (Drüeke and Massy, 2013). Cleavage of the intracellular domain of the full-length protein is performed by secretases—more specifically by α- β-secretase. Klotho is cleaved by α-secretases ADAM10 and 17 (A Disintegrin and Metalloprotease), as well as β-secretase BACE1 (Beta-Secretase 1). The remaining membrane-bound fragment is a substrate for regulated intramembrane proteolysis by γ-secretase (Bloch et al., 2009). Both of these processes lead to “soluble” or circulating Klotho, which is found in blood, urine, and cerebrospinal fluid (Matsumura et al., 1998; Wang and Sun, 2009; Drüeke and Massy, 2013). Higher concentrations of Klotho are associated with a slower aging process and fewer negative-health outcomes (Kuro-o et al., 1997; Kurosu et al., 2005; Xu and Sun, 2015). Evidence has shown Klotho to be lower in adults with obesity compared to their normal-weight counterparts (Amitani et al., 2013). This may suggest that treatment of overweight and obesity through weight loss may increase Klotho concentration, potentially counteracting the accelerated aging effects of obesity. However, the influence of intentional weight loss on Klotho concentration among adults with overweight or obesity has not been well characterized.

Behavioral interventions—the use of principles and techniques to change a participant’s behavior and habits (Wadden and Stunkard, 2002; Lang and Froelicher, 2006)—for weight loss are effective strategies for the treatment of overweight and obesity, with the majority of weight loss resulting from dietary changes via reduced energy intake (Dietary Guidelines Advisory Committee, 2015; Physical Activity Guidelines Advisory Committee, 2018; Heinicke and Halle, 2020). However, the addition of physical activity to dietary changes can enhance weight loss and result in additional health benefits compared to diet-induced weight loss alone (Webb and Wadden, 2017; Jakicic et al., 2018). The addition of physical activity to diet for weight loss may also have effects on Klotho concentration, as prior studies have shown an independent effect of physical activity increasing concentrations of Klotho (Avin et al., 2014). However, the effects of weight loss resulting from dietary changes alone or dietary changes in combination with physical activity have not been reported, warranting investigation.

The Heart Health Study aimed to examine the effect of a reduced calorie diet alone compared to diet in conjunction with one of two prescribed doses of physical activity on weight loss, measures of cardiac structure, and other cardiometabolic risk factors among adults with overweight or obesity (Jakicic et al., 2022). The Heart Health Study collected fasting blood samples, cardiorespiratory fitness, and body composition measures at baseline, 6 months, and 12 months during the weight loss intervention period. These data and blood samples were used to conduct this secondary analysis in a subsample of participants to examine: 1) the change in Klotho concentration in response a behavioral weight loss intervention by magnitude of weight loss achieved; and 2) the association among Klotho concentration and body weight, waist circumference, measures of body composition, and cardiorespiratory fitness.

## Materials and methods

**Study Design.** In the Heart Health Study (ClinicalTrials.gov NCT01500356, recruitment occurred between December 2011 and June 2015), participants completed assessments prior to (baseline), during (6 months), and at the end of a 12-month behavioral weight loss intervention. Participants were randomized to one of three intervention groups: 1) DIET—diet alone; 2) DIET + PA150—diet combined with progression to 150 min per week of prescribed moderate-to-vigorous intensity physical activity (MVPA), or 3) DIET + PA250—diet combined with progression to 250 min per week of prescribed MVPA. As previously reported, randomization was stratified by sex and race (white or nonwhite) in randomly selected block sizes (Jakicic et al., 2022). The Heart Health Study protocol was approved by the institutional review board at the University of Pittsburgh.

**Participants.** The protocol for participant recruitment has been previously reported (Jakicic et al., 2022). Eligibility criteria have previously been reported and included an age between 18 and 55 years and body mass index between 25 and <40 kg/m^2^ (Rogers et al., 2020). Exclusion criteria included 1) self-reporting ≥60 min/week of structured MVPA 2) weight loss of ≥5% within the prior 6 months or a history of bariatric surgery; 3) history of cardiometabolic disease, diabetes mellitus, or cancer; 4) taking medication that could affect heart rate or blood pressure; 5) taking medication that could influence body weight; 6) treatment for psychological conditions that included medication or counseling; 7) currently pregnant, pregnant within the prior 6 months, or planning a pregnancy within the next 12 months; 8) planning on geographical relocation outside of the region within 12 months; 9) inability to comply with the components of the interventions; or (10) had a contraindication that would prohibit cardiac magnetic resonance imaging scanning. Participants provided written informed consent and medical clearance from their physician prior to engaging in this study.

Because Klotho is a blood biomarker, to be eligible for this secondary analysis the participant needed to have blood samples available for analysis from the baseline, 6-month, and 12-month assessment periods, along with other outcome measures of interest for these secondary analyses. Moreover, because the secondary analysis focused on examining potential differences for change in Klotho concentration at lower and higher magnitudes of weight loss, an *a priori* decision was made to only include participants in these secondary analyses if they achieved ≥10% weight loss at both 6 months and 12 months (classified as a “responder”) or achieved <5% weight loss at both 6 months and 12 months (classified as a “non-responder”).

**Intervention.** As previously described, participants were randomized into DIET, DIET + PA150, and DIET + PA250 intervention groups for a period of 12 months (Jakicic et al., 2022). Participants in all intervention groups were instructed to attend weekly weight loss group sessions for weeks 1-24. For weeks 25-52 participants were instructed to attend in-person intervention sessions approximately every other week and to also receive an individual brief telephone intervention approximately every other week. If a participant missed a group session, a brief individual make-up session was offered to allow the content to be shared with the participant.

DIET, DIET + PA150, and DIET + PA250 were prescribed the same diet to reduce energy intake to be between 1,200 and 1,800 kcal/day based on baseline body weight, and to reduce dietary fat intake to be between 20% and 30% of total daily energy intake (Jakicic et al., 2022). The intervention staff reviewed self-monitoring records of dietary intake and provided written feedback to the participants.

Randomization groups differed in their prescribed physical activity (Jakicic et al., 2022). DIET was instructed to maintain their current level of physical activity and was not provided a prescription to increase their physical activity. DIET + PA150 was prescribed a progression to 150 min/week of unsupervised MVPA, whereas DIET + PA250 was prescribed a progression to 250 min/week of unsupervised MVPA.

**Demographic Characteristics.** Information on sex, race, and ethnicity were collected via questionnaire. Age was confirmed from the birth date listed on a government issued identification card (e.g., driver’s license or passport).

**Height, Weight, and Body Mass Index.** Weight and height were collected at baseline, 6 months, and 12 months (Rogers et al., 2020; Jakicic et al., 2022). Participants were clothed in a lightweight hospital gown and their shoes removed. Weight was assessed to the nearest 0.1 kg with duplicate measures differing by ≤ 0.2 kg using a calibrated digital scale. Height was assessed to the nearest 0.1 cm with duplicate measures differing by ≤ 0.5 cm using a wall-mounted stadiometer. Weight and height were used to calculate body mass index (BMI, kg/m^2^).

**Body Composition.** Body composition measures were assessed prior to the start of the intervention, 6 months, and 12 months (Jakicic et al., 2022). Participants were clothed in a lightweight hospital gown and their shoes removed. Women completed a urine pregnancy test to confirm non-pregnancy prior to the measurement. Total body composition, including measures of fat mass, lean mass, and percent body fat, were measured using dual-energy x-ray absorptiometry (DXA, GE Lunar iDXA, Madison, WI). Waist circumference was measured horizontally in duplicate at the iliac crest.

**Cardiorespiratory Fitness.** Submaximal cardiopulmonary exercise tests were conducted on all participants prior to the start of the intervention period, 6 months, and 12 months (Jakicic et al., 2022). All tests were performed using a motorized treadmill, with oxygen consumption measured using a calibrated metabolic cart. The test was terminated when the participant first achieved or exceeded 85% of their age-predicted maximal heart rate (HR_max_ = 220—age). Cardiorespiratory fitness is expressed in absolute (L/min) and relative terms (mL/kg/min). Change in cardiorespiratory fitness was computed as the difference between these values on the baseline test and on the subsequent 6-month and 12-month tests. Time to termination was measured as the time the cardiopulmonary exercise test was stopped in minutes.

**Blood Collection and Klotho Concentration.** Blood samples were collected at baseline, 6 months, and 12 months. Samples were collected in the morning with participants instructed to fast with the exception of water, abstain from exercise, and abstain from alcohol and smoking for at least 12 h. Blood samples were collected into evacuated tubes, processed in a refrigerated centrifuge, and stored at −80°C. For this secondary analysis, stored serum blood samples were used to measure Klotho concentration. Klotho was measured in duplicate by a solid-phase sandwich enzyme-linked immunosorbent assay (ELISA) (Yamazaki et al., 2010), with intra- and inter-assay coefficients of variation determined for the specific Klotho assays. The measurement procedure was performed as follows.

1) Test sample blank wells were determined, and 100 µL of EIA buffer was placed into the wells.
2) 100 µL of prepared test samples and 100 µL of prepared standard were placed into appropriate wells.
3) An incubation period of 60 min with the plate lid was performed at room temperature.
4) The plate was washed with the prepared wash buffer four times, with all liquid completely removed following the fourth wash.
5) 100 µL of the prepared labeled antibody was added into the wells.
6) An incubation period of 30 min with the plate lid was performed at room temperature.
7) The plate was washed with the prepared wash buffer five times, with all liquid completely removed follow the fifth wash.
8) 100 µL of Chromogen—TMB solution was added into the wells.
9) An incubation period of 30 min in the dark was performed at room temperature.
10) 100 µL of the Stop solution was added to the wells.
11) Removal of dirt and drops of water on the bottom of the plate was done, as well as confirmation that no bubbles were on the surface of the liquid. Then the optical density of the standard and the test samples were measured against a test sample blank, with the measurement wavelength at 450 nm. The minimum level of detectability of the assay was 6.15 pg/mL.

**Statistical Analysis.** Statistical analyses were performed using Statistical Package for the Social Sciences (SPSS) software, version 27. Repeated measures analyses of covariance (ANCOVA’s) modeled as *weight loss response category x time* were conducted to 1) assess the effect of the 12-month behavioral weight loss intervention by weight loss response (“responder” vs “non-responder”) on key variables of body weight, waist circumference, body composition, and cardiorespiratory fitness; and 2) to examine the change in Klotho across the 12-month behavioral weight loss intervention by weight loss response (“responder” vs “non-responder”). ANCOVA’s controlled for race and sex, which were randomization stratification variables for the parent study, intervention group, intervention group × time interaction, and baseline value of the variable being analyzed. Association between baseline Klotho concentration and baseline weight, waist circumference, body composition, and cardiorespiratory fitness, as well as changes from baseline to 6 months and 12 months were examined using the Spearman’s rho controlled for race and sex. The analyses assessing the association between the change in Klotho concentration at 6 or 12 months and the corresponding change in variables for weight, waist circumference, body composition, and cardiorespiratory fitness controlled for race, sex, intervention group, baseline Klotho concentration, and baseline value of the other corresponding variable. Statistical significance was defined at *p* ≤ 0.05. Because the outcome variable in the present study was not the primary outcome variable for the Heart Health Study, there were no *a priori* power calculations for this secondary analysis.

## Results

The parent study recruited and randomized 383 participants. As described above, a subsample of 152 participants (39.7% of the full sample) were classified as a Responder or Non-Responder, as well as provided blood samples at baseline, 6 months, and 12 months were included in these secondary analyses. Descriptive characteristics of the sample from the parent study and the subsample used for these analyses are shown in Table 1. Overall, participants included in this secondary analysis were 45.4 ± 8.0 years of age, women (77.6%), and White/Caucasian (76.3%). The distribution by intervention group is shown in Table 2.

**TABLE 1:** Baseline characteristics by sample.

Variable	Total sample (N = 383)		Sample with complete blood samples for klotho analysis (N = 152)
Age (years)	45.0 ± 7.9		45.4 ± 8.0
Weight (kg)	90.9 ± 13.7		90.4 ± 13.1
Body Mass Index (kg/m^2^)	32.4 ± 3.8		32.1 ± 3.7
Lean Mass (kg)	48.6 ± 8.7		48.1 ± 8.7
Fat Mass (kg)	39.2 ± 8.2		39.1 ± 7.8
Tissue Percent Body Fat (%)	44.5 ± 5.6		44.8 ± 5.6
Region Percent Body Fat (%)	43.2 ± 5.5		43.5 ± 5.6
Waist Circumference (cm)	106.4 ± 9.9		106.6 ± 9.6
Cardiorespiratory Fitness (mL/kg/min)	22.6 ± 4.4		22.9 ± 4.5
Cardiorespiratory Fitness (Termination Time - minutes)	7.7 ± 3.0		8.0 ± 3.0
Female (N, %)	304, 79.4%		118, 77.6%
Non-White (N, %)	104, 27.2%		36, 23.7%

Values are mean ± standard deviation unless indicated otherwise.

**TABLE 2:** Number of participants by randomized intervention group by weight loss response.

Classification	Intervention group		
	DIET	DIET + PA150	DIET+250
**N = 182**			
Responder	43	44	44
Non-Responder	20	15	16
N = 152*			
Responder	37	35	39
Non-Responder	15	13	13

*Indicates that blood samples were available for Klotho analysis at 0, 6, and 12 months. Responder: ≥10% weight loss at both 6 months and 12 months. Non-responder: <5% weight loss at both 6 months and 12 months.

In this subsample of participants, the 12-month behavioral weight loss intervention resulted in significantly greater reductions in body weight, BMI, lean mass, fat mass, percent body fat, and waist circumference (*p* < 0.0001) in Responders compared to Non-Responders. Moreover, the improvement in cardiorespiratory fitness (*p* < 0.0001) was significantly greater in Responders vs Non-Responders (Table 3).

**TABLE 3:** Change in Klotho (pg/mL), body weight (kg), lean mass (kg), fat mass (kg), and relative cardiorespiratory fitness (mL/kg/min) by weight loss response category (responder vs non-responder) across the 12-month intervention.

Variable	Category	Baseline mean (95% CI)	Change from baseline*		*p*-values		
			6 Month	12 Month	Category	Time***	Category X time***
Body Weight (kg)	Responder (N = 111)	91.0 (88.6, 93.4)	−14.1 (−14.8, −13.5)^**^	−16.8 (−17.8, −15.8)^**^	**<0.001**	0.236	**<0.001**
	Non-Responder (N = 41)	88.7 (84.8, 92.6)	−2.0 (−3.1, −0.9)^**^	−0.4 (−1.9, 1.1)			
Body Mass Index (kg/m^2^)	Responder (N = 111)	91.0 (88.6, 93.4)	−5.0 (−5.2, −4.8)^**^	−5.9 (−6.3, −5.6)^**^	**<0.001**	0.079	**<0.001**
	Non-Responder (N = 41)	88.7 (84.8, 92.6)	−0.7 (−1.0, −0.3)^**^	−0.1 (−0.7, 0.4)			
Lean Mass (kg)	Responder (N = 111)	48.4 (46.8, 49.9)	−2.2 (−2.1, −1.9)^**^	−2.3 (−2.6, −1.9)^**^	**<0.001**	0.205	0.762
	Non-Responder (N = 41)	47.2 (44.7, 49.8)	−0.1 (−0.5, 0.4)	−0.2 (−0.7, 0.4)			
Fat Mass (kg)	Responder (N = 111)	39.3 (37.9, 40.9)	−11.6 (−12.1, −11.0)^**^	−14.0 (−14.8, −13.2)^**^	**<0.001**	0.472	**<0.001**
	Non-Responder (N = 41)	38.4 (36.2, 40.7)	−2.0 (−2.8, −1.1)^**^	−0.3 (−1.7, 1.0)			
Percent Body Fat (%)	Responder (N = 111)	44.8 (43.7, 45.8)	−7.5 (−7.9, −7.0)^**^	−9.6 (−10.3, −8.9)^**^	**<0.001**	0.688	**<0.001**
	Non-Responder (N = 41)	45.0 (43.1, 46.4)	−1.2 (−2.0, −0.5)^**^	−0.1 (−1.3, 1.0)			
Waist Circumference (cm)	Responder (N = 111)	107.3 (105.4, 109.2)	−12.6 (−13.5, −11.6)^**^	−15.2 (−16.3, −14.1)^**^	**<0.001**	0.185	**0.010**
	Non-Responder (N = 41)	104.5 (101.7, 107.3)	−2.0 (−3.6, −0.3)^**^	−2.2 (−4.1, −0.3)^**^			
Cardiorespiratory Fitness (mL/kg/min)	Responder (N = 110)	23.0 (22.3, 23.8)	3.9 (3.3, 4.5)^**^	4.7 (4.1, 5.3)^**^	**<0.001**	0.154	**<0.001**
	Non-Responder (N = 37)	22.6 (21.2, 24.2)	0.8 (−0.1, 1.8)	−0.5 (−1.6, 0.6)			
Cardiorespiratory Fitness (minute to termination)	Responder (N = 111)	8.0 (7.5, 8.6)	2.9 (2.5, 3.3)^**^	2.9 (2.5, 3.3)^**^	**<0.001**	0.556	0.099
	Non-Responder (N = 40)	7.9 (7.0, 8.8)	0.8 (0.1, 1.5)^**^	0.0 (−0.7, 0.7)			
Klotho (pg/mL)	Responder (N = 111)	936.2 (870.6, 1006.5)	74.1 (30.5, 117.7)^**^	25.9 (−16.7, 68.5)	**0.047**	0.547	0.727
	Non-Responder (N = 41)	926.1 (800.4, 1077.0)	−10.6 (−84.1, 62.9)	−44.7 (−116.6, 27.1)			

*LS Means (95% confidence interval); analysis adjusted for race, sex, intervention group, and baseline value.

**p < 0.05 for change from baseline.

***Time effect represents the comparison of change scores at 6 and 12 months.

Bolded values indicate statistical significance (*p* < 0.05).

**FIGURE 1:**
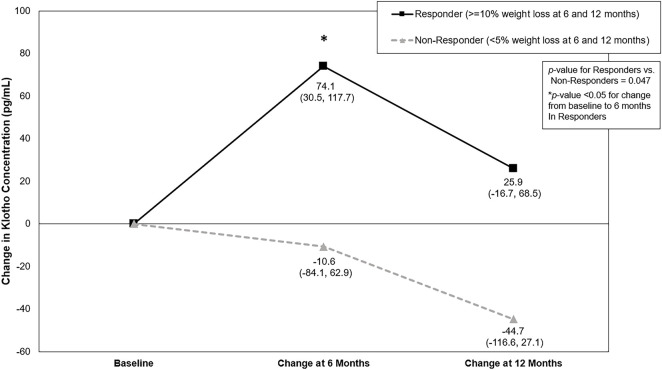
Change in Klotho concentration (pg/mL) by weight loss response category across the 12-month behavioral weight loss intervention. Results based on analysis of covariance adjusted for race, sex, intervention group, and baseline Klotho concentration. Values represent LS means (95% confidence interval).

Baseline Klotho concentration for Responders was 936.2 pg/mL (95% Confidence Interval (CI): 870.6, 1006.5) and 926.1 pg/mL (95% CI: 800.4, 1077.0) for Non-Responders. There was a significant difference for the change in Klotho from baseline between Responders and Non-Responders (Baseline to 6-month change: 74.1 (95% CI: 30.5, 117.7) pg/mL vs. −10.6 (95% CI: −84.1, 62.9) pg/mL; Baseline to 12-month change: 25.9 (95% CI: −16.7, 68.5) pg/mL vs. −44.7 (95% CI: −116.6, 27.1) pg/mL; *p* = 0.047) (Table 3; Figure 1). Klotho concentration significantly increased from baseline to 6 months in Responders; however, while Klotho remained above baseline levels at 12 months, the change from baseline to 12 months was not statistically significant. The change in Klotho concentration from baseline to both 6 and 12 months for Non-Responders was not statistically significant. Of potential importance, when intervention group (DIET, DIET + PA150, and DIET + PA250) was entered into the ANCOVA, intervention group as a covariate was not statistically significant.

Correlation analysis, controlling for covariates described above, showed baseline Klotho concentration was not significantly associated with measures of weight, body composition, waist circumference, or cardiorespiratory fitness (Table 4). The change in Klotho concentration from baseline to 6 months was inversely associated with change in weight (*r*_s_ = −0.195, *p* = 0.019), BMI (*r*_s_ = −0.196, *p* = 0.016), fat mass (*r*_s_ = −0.184, *p* = 0.026), and waist circumference (*r*_s_ = −0.218, *p* = 0.008) but not significantly associated with the change in other measures of body composition or cardiorespiratory fitness. The change in Klotho from baseline to 12 months was not significantly associated with the change in measures of weight, body composition, waist circumference, or cardiorespiratory fitness.

**TABLE 4:** Spearman’s rho between Klotho concentration at baseline and change at 6- and 12 months with corresponding measures of body weight, body mass index, body composition, and cardiorespiratory fitness.

Variable	Assessment period	Klotho concentration (pg/mL)		
		Baseline*	Change^#^: Baseline to 6 months**	Change^#^: Baseline to 12 months**
Body Weight (kg) [N = 152]	Baseline	r_s_ = −0.076 (*p* = 0.361)	-----	-----
	Change^#^: baseline to 6 months	-----	**r = -0.223 (*p* = 0.007)**	-----
	Change^#^: baseline to 12 months	-----	-----	r = −0.070 (*p* = 0.399)
Body Mass Index (kg/m^2^) [N = 152]	Baseline	r_s_ = −0.100 (*p* = 0.226)	-----	-----
	Change^#^: baseline to 6 months	-----	**r = -0.205 (*p* = 0.013)**	-----
	Change^#^: baseline to 12 months	-----	-----	r = −0.068 (*p* = 0.412)
Lean Mass (kg) [N = 152]	Baseline	r_s_ = −0.148 (*p* = 0.073)	-----	-----
	Change^#^: baseline to 6 months	-----	r = −0.108 (*p* = 0.195)	-----
	Change^#^: baseline to 12 months	-----	-----	r = −0.122 (*p* = 0.143)
Fat Mass (kg) [N = 152]	Baseline	r_s_ = 0.005 (*p* = 0.952)	-----	-----
	Change^#^: baseline to 6 months	-----	***r* = -0.192 (*p* = 0.020)**	-----
	Change^#^: baseline to 12 months	-----	-----	r = −0.042 (*p* = 0.615)
Percent Body Fat (%) [N = 152]	Baseline	r_s_ = 0.067 (*p* = 0.421)	-----	-----
	Change^#^: baseline to 6 months	-----	*r* = −0.188 (*p* = 0.023)	-----
	Change^#^: baseline to 12 months	-----	-----	r = −0.061 (*p* = 0.464)
Waist Circumference (cm) [N = 152]	Baseline	*r*_s_ = −0.079 (*p* = 0.340)	-----	-----
	Change^#^: baseline to 6 months	-----	***r***_**s**_ **= -0.218 (*p* = 0.008)**	-----
	Change^#^: baseline to 12 months	-----	-----	r_s_ = −0.026 (*p* = 0.752)
Cardiorespiratory Fitness (mL/kg/min) [N = 147]	Baseline	*r*_s_ = 0.015 (*p* = 0.856)	-----	-----
	Change^#^: baseline to 6 months	-----	*r* = 0.042 (*p* = 0.620)	-----
	Change^#^: baseline to 12 months	-----	-----	*r* = 0.067 (*p* = 0.424)
Cardiorespiratory Fitness (Termination Time, minutes) [N = 151]	Baseline	*r*_s_ = 0.036 (*p* = 0.660)	-----	-----
	Change^#^: baseline to 6 months	-----	*r* = 0.020 (*p* = 0.814)	-----
	Change^#^: baseline to 12 months	-----	-----	*r* = 0.102 (*p* = 0.221)

*Analysis controlled for race and sex.

**Analysis controlled for race, sex, intervention condition, baseline Klotho concentration, and baseline of other corresponding variable.

^#^6-month change = 6-month value minus baseline value; 12-month change = 12-month value minus baseline

Bolded values indicate statistical significance (*p* < 0.05).

## Discussion

The purpose of this secondary analysis was to investigate the potential association between obesity, weight loss, and Klotho concentration. Obesity is associated with many chronic conditions and promotion of advanced aging activity (Ahima, 2009; Barton, 2010), with Klotho being a biomarker of premature aging processes (Kuro-o et al., 1997; Xu and Sun, 2015). We found Klotho concentration significantly increased from baseline to 6 months in participants with weight loss ≥10% (Responders), but the increase observed at 12 months was no longer statistically significant. The Non-Responders had no significant change in Klotho concentration at either 6 or 12 months.

Baseline Klotho concentration for the middle-aged adults with overweight or obesity included in this secondary analysis was approximately 930 pg/mL. However, there is variability surrounding the concentration of circulating Klotho dependent upon age and BMI. Amitani et al. (2013), investigated Klotho’s role in human metabolism by examining the association between plasma Klotho concentration and BMI. Participants (*n* = 34) either with normal weight (mean BMI: 21.8 kg/m^2^), underweight or diagnosed with anorexia nervosa (mean BMI: 13.1 kg/m^2^), or with obesity (mean BMI: 35.7 kg/m^2^) and a mean age of 21 years old. Findings show mean Klotho concentration for participants with normal weight to be 1391.6 pg/mL, underweight or diagnosed with anorexia nervosa to be 764.6 pg/mL, and for participants with obesity to be 847.1 pg/mL (Amitani et al., 2013). When studying children and adolescents of ranging BMI’s, Klotho concentrations continue in this variable trend. Children and adolescents with obesity had a significantly higher Klotho concentration (median: 168.6 pg/mL) compared to individuals with overweight (median: 131.3 pg/mL) and normal weight status (median: 116.6 pg/mL) (Socha-Banasiak et al., 2020). The change in Klotho concentration, to the best of our knowledge, has not been reported for healthy young and middle-aged adults with overweight or obesity undergoing a behavioral weight loss intervention. The findings presented in this report suggest that weight loss, of sufficient magnitude, may have a favorable influence on this biomarker of aging, which may be of clinical importance. This may also suggest that in adults with excess weight or obesity, intentional weight loss may have a favorable influence of reducing the risk for premature deleterious health effects of aging. There is evidence that Klotho, specifically β-Klotho, acts as an essential component in endocrine fibroblast growth factor receptor complexes. β-Klotho is required for high-affinity binding of endocrine fibroblast growth factors to induce a signaling cascade (Kuro-o, 2019). This cascade is actively involved in homeostatic maintenance of glucose metabolism,—which is largely dependent on adipose tissue—energy expenditure, and cardiovascular complications in abdominal obesity and obesity-related diseases (Chen et al., 2007; Ornitz and Itoh, 2015; Scheja and Heeren, 2016; Meex and Watt, 2017; Ghadge et al., 2018). Potentially signifying Klotho concentrations may serve as a predictor for prediabetes, type 2 diabetes, cardiovascular disease, and obesity, however more work needs to be done in this area of research.

Abdominal obesity, or visceral obesity, is independently linked to several pathological conditions including impaired glucose and lipid metabolism, insulin resistance, increased predisposition to cancers of the colon, breast, and prostate, prolonged hospital stays, increased incidence of infections and non-infectious complications, and increased mortality in the hospital (Schapira et al., 1994; Von Hafe et al., 2004; Fox et al., 2007; Ritchie and Connell, 2007; Oh et al., 2008; Tsujinaka et al., 2008). The similar pathological pathways affected by visceral adiposity and Klotho suggest a possible relationship. A cross-sectional analysis aimed to evaluate the association between visceral adiposity index and serum Klotho among 6,252 adults (Cui et al., 2023). The visceral adiposity index represents the waist circumference relative to the combination of BMI, triglycerides, and high-density lipoprotein. A multivariable regression analysis found serum Klotho concentration was lower in participants with a high visceral adiposity index. A segmented regression analyses showed that this relationship was non-linear, only being observed when the visceral adiposity index score was less than 3.21. These findings may suggest that a lower level of adiposity may have anti-aging and health benefits by increasing Klotho concentrations (Cui et al., 2023). However, this analysis is limited in its cross-sectional nature and suggests the need for prospective studies to confirm a causal relationship and mechanistic pathway. The current study demonstrated a significant association between reduction in waist circumference at 6 months and the change in Klotho, suggesting that reduced abdominal adiposity may contribute to beneficial changes in biomarkers reflective of healthy aging.

While Klotho concentration did significantly increase at 6 months in Responders—defined as reducing weight by at least 10%—the increase in Klotho concentration from baseline to 12 months in these participants was no longer statistically significant despite weight loss being sustained. This may suggest that Klotho begins to regress toward baseline levels during periods of weight loss maintenance. Of interest, the regression of health benefits observed with weight loss has been shown in response to other weight loss treatments. A randomized clinical trial from Courcoulas and others (Courcoulas et al., 2015), observed significant weight loss among individuals who underwent bariatric surgery, which was maintained up to 3 years following the intervention (Courcoulas et al., 2015). Moreover, 60% individuals who underwent Roux-en-Y Gastric Bypass (RYGB) surgery and 45% of individuals who underwent laparoscopic adjustable gastric band (LAGB) surgery had complete or partial remission of type 2 diabetes at year one following surgery. However, 3 years following surgery only 29% of the RYGB and 40% of the LAGB participants remained in remission (Courcoulas et al., 2015). Thus, the pattern of change in Klotho concentration observed in the current study may not be a unique phenomenon, but rather the body’s potential desire to return to baseline levels despite weight loss maintenance and this warrants further investigation.

There is evidence to support performing exercise may lead to improvements in Klotho concentration in both human and animal models (Avin et al., 2014; Dalise et al., 2017); potentially indicating reducing sedentary time and increasing muscle activity through exercise may provide either improvements or maintenance in Klotho concentration. In a 16-week exercise training program (Avin et al., 2014) among individuals with obesity, participants showed a substantial increase in Klotho concentration in response to an acute exercise bout performed post-intervention compared to an acute exercise bout performed pre-intervention (Avin et al., 2014). Further, in mice models (Dalise et al., 2017), a 30-min bout of exercise resulted in a significant upregulation of Klotho in mice from pre-to post-exercise bout (Dalise et al., 2017). However, when adults with overweight or obesity undergo a behavioral weight loss intervention using dietary restriction and two intervention groups including physical activity, the association between change in Klotho concentration and weight loss was not altered. Moreover, change in Klotho concentration was not significantly associated with change in cardiorespiratory fitness. This may suggest that the effects of physical activity, or physical activity that increases cardiorespiratory fitness, on Klotho concentration may be influenced by whether physical activity is accompanied with dietary restriction that results in weight loss. This potential difference in response warrants further investigation.

Strengths include that the parent study included a 12-month behavioral weight loss intervention, with measures collected prior to, during, and post-intervention. This intervention resulted in variability in weight loss, that allowed the present analysis to investigate change in Klotho concentration in participants with at least 10% weight loss (Responders) compared to participants with less than 5% weight loss (Non-Responders).

Despite these strengths, the present analysis is not without limitations that could impact the interpretation of the observed results. The study sample included individuals with overweight or obesity but otherwise relatively healthy individuals, therefore functional impairments needed to observe associations with Klotho concentrations may not have been detected. This analysis contains a subsample of the original 383 participants recruited for the parent Heart Health Study, with only 152 participants included and stratified into category of weight loss response classification to the weight loss intervention. Though weight loss of ≥10% is clinically meaningful, whether Klotho concentration changes differently with other magnitudes of weight loss may warrant future research. Also, the average age of this population was 45 years, representing a slightly younger population than what has been typically reported within the Klotho literature. Thus, future research should consider assessing the impact of intentional weight loss on Klotho concentration among older adults, where Klotho concentration has been predominantly studied, and where excess body weight and adiposity are also present. Although Klotho is primarily produced and released into circulation by the kidney (Kim et al., 2018; Cheikhi et al., 2019), this study did not measure or estimate renal function. Klotho levels are strongly correlated with chronic kidney disease and renal failure, and individuals with obesity are at a greater risk for developing these conditions (Kim et al., 2018; Lakkis and Weir, 2018; Nehus, 2018; Cheikhi et al., 2019; Chen et al., 2021). Therefore, future research should consider including a measure of renal function in their screening criteria.

In conclusion, this secondary analysis of the Heart Health Study provides evidence within the context of a behavioral weight loss intervention, that Klotho concentration significantly increases with weight loss of ≥10%. This may suggest that weight loss in middle-aged adults with overweight or obesity may have a favorable effect on biomarkers associated with aging, and this warrants additional investigation. Moreover, future studies should examine whether similar findings are present in older adults with overweight or obesity and whether weight loss has a similar effect as what was observed in this current study. There is also a need for future studies to disentangle the potential effects of variations in macro-nutrient composition. These findings may have clinical importance for understanding the effects of excess weight and adiposity, along with reductions in these factors, on health outcomes.

## Data Availability

The raw data supporting the conclusion of this article will be made available by the authors, without undue reservation, upon reasonable request.

## References

[B1] Ahima R. S. (2009). Connecting obesity, aging and diabetes. Nat. Med. 15, 996–997. 10.1038/nm0909-996 19734871

[B2] Amitani M. Asakawa A. Amitani H. Kaimoto K. Sameshima N. Koyama K. I. (2013). Plasma klotho levels decrease in both anorexia nervosa and obesity. Nutrition 29, 1106–1109. 10.1016/j.nut.2013.02.005 23790542

[B3] Arking D. E. Becker D. M. Yanek L. R. Fallin D. Judge D. P. Moy T. F. (2003). KLOTHO allele status and the risk of early-onset occult coronary artery disease. Am. J. Hum. Genet. 72, 1154–1161. 10.1086/375035 12669274 PMC1180268

[B4] Avin K. G. Coen P. M. Huang W. Stolz D. B. Sowa G. A. Dubé J. J. (2014). Skeletal muscle as a regulator of the longevity protein, Klotho. Front. Physiology 5, 189. 10.3389/fphys.2014.00189 PMC4060456 24987372

[B5] Barton M. (2010). Obesity and aging: Determinants of endothelial cell dysfunction and atherosclerosis. Pflügers Archiv-European J. Physiology 460, 825–837. 10.1007/s00424-010-0860-y 20635093

[B6] Bloch L. Sineshchekova O. Reichenbach D. Reiss K. Saftig P. Kuro-O M. (2009). Klotho is a substrate for alpha-beta- and gamma-secretase. FEBS Lett. 583, 3221–3224. 10.1016/j.febslet.2009.09.009 19737556 PMC2757472

[B7] Cheikhi A. Barchowsky A. Sahu A. Shinde S. N. Pius A. Clemens Z. J. (2019). Klotho: An elephant in aging research. J. Gerontol. A Biol. Sci. Med. Sci. 74, 1031–1042. 10.1093/gerona/glz061 30843026 PMC7330474

[B8] Chen C.-D. Podvin S. Gillespie E. Leeman S. E. Abraham C. R. (2007). Insulin stimulates the cleavage and release of the extracellular domain of Klotho by ADAM10 and ADAM17. Proc. Natl. Acad. Sci. 104, 19796–19801. 10.1073/pnas.0709805104 18056631 PMC2148378

[B9] Chen Y. Dabbas W. Gangemi A. Benedetti E. Lash J. Finn P. W. (2021). Obesity management and chronic kidney disease. Semin. Nephrol. 41, 392–402. 10.1016/j.semnephrol.2021.06.010 34715968

[B10] Consitt L. A. Bell J. A. Houmard J. A. (2009). Intramuscular lipid metabolism, insulin action, and obesity. IUBMB Life 61, 47–55. 10.1002/iub.142 18839419 PMC2612735

[B11] Courcoulas A. P. Belle S. H. Neiberg R. H. Pierson S. K. Eagleton J. K. Kalarchian M. A. (2015). Three-year outcomes of bariatric surgery vs lifestyle intervention for type 2 diabetes mellitus treatment: A randomized clinical trial. J. Am. Med. Assoc. Surg. 150, 931–940. 10.1001/jamasurg.2015.1534 PMC4905566 26132586

[B12] Cui J. Yang Z. Wang J. Yin S. Xiao Y. Bai Y. (2023). A cross-sectional analysis of association between visceral adiposity index and serum anti-aging protein Klotho in adults. Front. Endocrinol. 14, 1082504. 10.3389/fendo.2023.1082504 PMC9939517 36814582

[B13] Dalise S. Cavalli L. Ghuman H. Wahlberg B. Gerwig M. Chisari C. (2017). Biological effects of dosing aerobic exercise and neuromuscular electrical stimulation in rats. Sci. Rep. 7, 10830. 10.1038/s41598-017-11260-7 28883534 PMC5589775

[B14] Di Bona D. Accardi G. Virruso C. Candore G. Caruso C. (2014). Association of klotho polymorphisms with healthy aging: A systematic review and meta-analysis. Rejuvenation Res. 17, 212–216. 10.1089/rej.2013.1523 24164579

[B15] DIETARY GUIDELINES ADVISORY COMMITTEE (2015). “2015-2020 dietary Guidelines for Americans,” in AGRICULTURE, U. S. D. O. H. A. H. S. A. U. S. D. O. (ed.) (Washington D.C).

[B16] Drüeke T. B. Massy Z. A. (2013). Circulating klotho levels: Clinical relevance and relationship with tissue klotho expression. Kidney Int. 83, 13–15. 10.1038/ki.2012.370 23271484

[B17] Field A. E. Coakley E. H. Must A. Spadano J. L. Laird N. Dietz W. H. (2001). Impact of overweight on the risk of developing common chronic diseases during a 10-year period. Archives Intern. Med. 161, 1581–1586. 10.1001/archinte.161.13.1581 11434789

[B18] Flegal K. M. Carroll M. D. Kit B. K. Ogden C. L. (2012). Prevalence of obesity and trends in the distribution of body mass index among US adults, 1999-2010. J. Am. Med. Assoc. 307, 491–497. 10.1001/jama.2012.39 22253363

[B19] Fox C. S. Massaro J. M. Hoffmann U. Pou K. M. Maurovich-Horvat P. Liu C.-Y. (2007). Abdominal visceral and subcutaneous adipose tissue compartments: Association with metabolic risk factors in the framingham heart study. Circulation 116, 39–48. 10.1161/CIRCULATIONAHA.106.675355 17576866

[B20] Galgani J. Ravussin E. (2009). Energy metabolism, fuel selection and body weight regulation. Int. J. Obes. 32, S109–S119. 10.1038/ijo.2008.246 PMC2897177 19136979

[B21] Ghadge A. A. Khaire A. A. Kuvalekar A. A. (2018). Adiponectin: A potential therapeutic target for metabolic syndrome. Cytokine Growth Factor Rev. 39, 151–158. 10.1016/j.cytogfr.2018.01.004 29395659

[B22] Hales C. M. Carroll M. D. Fryar C. D. Ogden C. L. (2017). Prevalence of obesity among adults and youth: United States, 2015-2016, US department of health and human services, centers for disease control and prevention. National Center for Health Statistics.

[B23] He J. Watkins S. Kelley D. E. (2001). Skeletal muscle lipid content and oxidative enzyme activity in relation to muscle fiber type in type 2 diabetes and obesity. Diabetes 50, 817–823. 10.2337/diabetes.50.4.817 11289047

[B24] Heinicke V. Halle M. (2020). Lifestyle intervention in the primary prevention of cardiovascular diseases. Herz 45, 30–38. 10.1007/s00059-019-04886-y 31993680

[B25] Hui H. Zhai Y. Ao L. Cleveland J. R. J. C. Liu H. Fullerton D. A. (2017). Klotho suppresses the inflammatory responses and ameliorates cardiac dysfunction in aging endotoxemic mice. Oncotarget 8, 15663–15676. 10.18632/oncotarget.14933 28152512 PMC5362514

[B26] Jakicic J. M. Rogers R. J. Davis K. K. Collins K. A. (2018). Role of physical activity and exercise in treating patients with overweight and obesity. Clin. Chem. 64, 99–107. 10.1373/clinchem.2017.272443 29158251

[B27] Jakicic J. M. Rogers R. J. Lang W. Gibbs B. B. Yuan N. Fridman Y. (2022). Impact of weight loss with diet or diet plus physical activity on cardiac magnetic resonance imaging and cardiovascular disease risk factors: Heart Health Study randomized trial. Obesity 30, 1039–1056. 10.1002/oby.23412 35470972 PMC9813917

[B28] Kim H. J. Kang E. Oh Y. K. Kim Y. H. Han S. H. Yoo T. H. (2018). The association between soluble klotho and cardiovascular parameters in chronic kidney disease: Results from the KNOW-ckd study. BMC Nephrol. 19, 51. 10.1186/s12882-018-0851-3 29506503 PMC5838864

[B29] Kuro-O M. Matsumura Y. Aizawa H. Kawaguchi H. Suga T. Utsugi T. (1997). Mutation of the mouse klotho gene leads to a syndrome resembling ageing. Nature 390, 45–51. 10.1038/36285 9363890

[B30] Kuro-O M. (2019). The Klotho proteins in health and disease. Nat. Rev. Nephrol. 15, 27–44. 10.1038/s41581-018-0078-3 30455427

[B31] Kurosu H. Yamamoto M. Clark J. D. Pastor J. V. Nandi A. Gurnani P. (2005). Suppression of aging in mice by the hormone Klotho. Science 309, 1829–1833. 10.1126/science.1112766 16123266 PMC2536606

[B32] Lakkis J. I. Weir M. R. (2018). Obesity and kidney disease. Prog. Cardiovasc Dis. 61, 157–167. 10.1016/j.pcad.2018.07.005 29981350

[B33] Lang A. Froelicher E. S. (2006). Management of overweight and obesity in adults: Behavioral intervention for long-term weight loss and maintenance. Eur. J. Cardiovasc. Nurs. 5, 102–114. 10.1016/j.ejcnurse.2005.11.002 16406709

[B34] Matsumura Y. Aizawa H. Shiraki-Iida T. Nagai R. Kuro-O M. Nabeshima Y.-I. (1998). Identification of the human klotho gene and its two transcripts encoding membrane and secreted klotho protein. Biochem. Biophysical Res. Commun. 242, 626–630. 10.1006/bbrc.1997.8019 9464267

[B35] Meex R. C. R. Watt M. J. (2017). Hepatokines: Linking nonalcoholic fatty liver disease and insulin resistance. Nat. Rev. Endocrinol. 13, 509–520. 10.1038/nrendo.2017.56 28621339

[B36] Mokdad A. H. Ford E. S. Bowman B. A. Dietz W. H. Vinicor F. Bales V. S. (2003). Prevalence of obesity, diabetes, and obesity-related health risk factors, 2001. J. Am. Med. Assoc. 289, 76–79. 10.1001/jama.289.1.76 12503980

[B37] Must A. Spadano J. Coakley E. H. Field A. E. Colditz G. Dietz W. H. (1999). The disease burden associated with overweight and obesity. J. Am. Med. Assoc. 282, 1523–1529. 10.1001/jama.282.16.1523 10546691

[B39] Nehus E. (2018). Obesity and chronic kidney disease. Curr. Opin. Pediatr. 30, 241–246. 10.1097/MOP.0000000000000586 29346138

[B40] Oh T.-H. Byeon J.-S. Myung S.-J. Yang S.-K. Choi K.-S. Chung J.-W. (2008). Visceral obesity as a risk factor for colorectal neoplasm. J. Gastroenterology Hepatology 23, 411–417. 10.1111/j.1440-1746.2007.05125.x 17725596

[B41] Orces C. H. (2022a). The association between metabolic syndrome and the anti-aging humoral factor klotho in middle-aged and older adults. Diabetes Metab. Syndr. 16, 102522. 10.1016/j.dsx.2022.102522 35660935

[B42] Orces C. H. (2022b). The association of obesity and the antiaging humoral factor klotho in middle-aged and older adults. ScientificWorldJournal 2022, 7274858. 10.1155/2022/7274858 36061981 PMC9433301

[B43] Ornitz D. M. Itoh N. (2015). The fibroblast growth factor signaling pathway. WIREs Dev. Biol. 4, 215–266. 10.1002/wdev.176 PMC4393358 25772309

[B44] PHYSICAL ACTIVITY GUIDELINES ADVISORY COMMITTEE (2018). “2018 physical activity Guidelines advisory committee scientific report,” in SERVICES, U. S. D. O. H. A. H. (Washington, D.C.

[B45] Poirier P. Giles T. D. Bray G. A. Hong Y. Stern J. S. Pi-Sunyer F. X. (2006). Obesity and cardiovascular disease: Pathophysiology, evaluation, and effect of weight loss: An update of the 1997 American heart association scientific statement on obesity and heart disease from the obesity committee of the council on nutrition, physical activity, and metabolism. Circulation 113, 898–918. 10.1161/CIRCULATIONAHA.106.171016 16380542

[B46] Razzaque M. S. (2012). The role of Klotho in energy metabolism. Nat. Rev. Endocrinol. 8, 579–587. 10.1038/nrendo.2012.75 22641000 PMC3704949

[B47] Ritchie S. A. Connell J. M. (2007). The link between abdominal obesity, metabolic syndrome and cardiovascular disease. Nutr. Metabolism, Cardiovasc. Dis. 17, 319–326. 10.1016/j.numecd.2006.07.005 17110092

[B48] Rogers R. J. Schelbert E. B. Lang W. Fridman Y. Yuan N. Jakicic J. M. (2020). Association of fitness and body fatness with left ventricular mass: The heart health study. Obes. Sci. Pract. 6, 19–27. 10.1002/osp4.380 32128239 PMC7042021

[B49] Schapira D. V. Clark R. A. Wolff P. A. Jarrett A. R. Kumar N. B. Aziz N. M. (1994). Visceral obesity and breast cancer risk. Cancer 74, 632–639. 10.1002/1097-0142(19940715)74:2<632::aid-cncr2820740215>3.0.co;2-t 8033042

[B50] Scheja L. Heeren J. (2016). Metabolic interplay between white, beige, Brown adipocytes and the liver. J. Hepatology 64, 1176–1186. 10.1016/j.jhep.2016.01.025 26829204

[B51] Seidell J. Muller D. Sorkin J. Andres R. (1992). Fasting respiratory exchange ratio and resting metabolic rate as predictors of weight gain: The baltimore longitudinal study on aging. Int. Journall Obes. Relat. Metabolic Disord. 16, 667–674. 1328091

[B52] Semba R. D. Cappola A. R. Sun K. Bandinelli S. Dalal M. Crasto C. (2011). Plasma klotho and cardiovascular disease in adults. J. Am. Geriatrics Soc. 59, 1596–1601. 10.1111/j.1532-5415.2011.03558.x PMC3486641 21883107

[B53] Semba R. D. Cappola A. R. Sun K. Bandinelli S. Dalal M. Crasto C. (2012). Relationship of low plasma klotho with poor grip strength in older community-dwelling adults: The InCHIANTI study. Eur. J. Appl. Physiology 112, 1215–1220. 10.1007/s00421-011-2072-3 PMC3435096 21769735

[B54] Semba R. D. Ferrucci L. Sun K. Simonsick E. Turner R. Miljkovic I. (2015). Low plasma klotho concentrations and decline of knee strength in older adults. Journals Gerontology Ser. A Biomed. Sci. Med. Sci. 71, 103–108. 10.1093/gerona/glv077 PMC4706099 26359247

[B55] Socha-Banasiak A. Michalak A. Pacześ K. Gaj Z. Fendler W. Socha A. (2020). Klotho and fibroblast growth factors 19 and 21 serum concentrations in children and adolescents with normal body weight and obesity and their associations with metabolic parameters. BMC Pediatr. 20, 294. 10.1186/s12887-020-02199-2 32546231 PMC7296965

[B56] Steinberg H. O. Chaker H. Leaming R. Johnson A. Brechtel G. Baron A. D. (1996). Obesity/insulin resistance is associated with endothelial dysfunction. Implications for the syndrome of insulin resistance. J. Clin. Investigation 97, 2601–2610. 10.1172/JCI118709 PMC507347 8647954

[B69] Stierman B. Afful J. Carroll M. D. Chen T.-C. Davy O. Fink S. (2021). “National health and nutrition examination survey 2017‐March 2020 prepandemic data files development of files and prevalence estimates for selected health outcomes,” in National Health Statistics Reports. Editor National Center for Health S. (Hyattsville, MD). 10.15620/cdc:106273 PMC11513744 39380201

[B57] Tsujinaka S. Konishi F. Kawamura Y. J. Saito M. Tajima N. Tanaka O. (2008). Visceral obesity predicts surgical outcomes after laparoscopic colectomy for sigmoid colon cancer. Dis. Colon and Rectum 51, 1757–1765. 10.1007/s10350-008-9395-0 18600376

[B58] Van Gaal L. F. Mertens I. L. Christophe E. (2006). Mechanisms linking obesity with cardiovascular disease. Nature 444, 875–880. 10.1038/nature05487 17167476

[B59] Vgontzas A. N. Papanicolaou D. A. Bixler E. O. Hopper K. Lotsikas A. Lin H.-M. (2000). Sleep apnea and daytime sleepiness and fatigue: Relation to visceral obesity, insulin resistance, and hypercytokinemia. J. Clin. Endocrinol. Metabolism 85, 1151–1158. 10.1210/jcem.85.3.6484 10720054

[B60] Von Hafe P. Pina F. Pérez A. Tavares M. Barros H. (2004). Visceral fat accumulation as a risk factor for prostate cancer. Obes. Res. 12, 1930–1935. 10.1038/oby.2004.242 15687393

[B61] Wadden T. A. Stunkard A. J. (2002). Handbook of obesity treatment. Guilford Press.

[B62] Wang Y. Sun Z. (2009). Current understanding of klotho. Ageing Res. Rev. 8, 43–51. 10.1016/j.arr.2008.10.002 19022406 PMC2637560

[B63] Webb V. L. Wadden T. A. (2017). Intensive lifestyle intervention for obesity: Principles, practices, and results. Gastroenterology 152, 1752–1764. 10.1053/j.gastro.2017.01.045 28192109

[B64] Weinsier R. L. Hunter G. R. Heini A. F. Goran M. I. Sell S. M. (1998). The etiology of obesity: Relative contribution of metabolic factors, diet, and physical activity. Am. J. Med. 105, 145–150. 10.1016/s0002-9343(98)00190-9 9727822

[B65] Wolk R. Shamsuzzaman A. S. Somers V. K. (2003). Obesity, sleep apnea, and hypertension. Hypertension 42, 1067–1074. 10.1161/01.HYP.0000101686.98973.A3 14610096

[B66] Woo K. Chook P. Yu C. Sung R. Qiao M. Leung S. (2004). Overweight in children is associated with arterial endothelial dysfunction and intima-media thickening. Int. J. Obes. 28, 852–857. 10.1038/sj.ijo.0802539 15170465

[B67] Xu Y. Sun Z. (2015). Molecular basis of klotho: From gene to function in aging. Endocr. Rev. 36, 174–193. 10.1210/er.2013-1079 25695404 PMC4399270

[B68] Yamazaki Y. Imura A. Urakawa I. Shimada T. Murakami J. Aono Y. (2010). Establishment of sandwich ELISA for soluble alpha-Klotho measurement: Age-dependent change of soluble alpha-Klotho levels in healthy subjects. Biochem. Biophysical Res. Commun. 398, 513–518. 10.1016/j.bbrc.2010.06.110 PMC4130489 20599764

